# Conservation of tandem stop codons in yeasts

**DOI:** 10.1186/gb-2005-6-4-r31

**Published:** 2005-03-15

**Authors:** Han Liang, Andre RO Cavalcanti, Laura F Landweber

**Affiliations:** 1 Department of Chemistry, Princeton University, Princeton, NJ 08544, USA; 2 Department of Ecology and Evolutionary Biology, Princeton University, Princeton, NJ 08544, USA

## Abstract

**Background:**

It has been long thought that the stop codon in a gene is followed by another stop codon that acts as a backup if the real one is read through by a near-cognate tRNA. The existence of such 'tandem stop codons', however, remains elusive.

**Results:**

Here we show that a statistical excess of stop codons has evolved at the third codon downstream of the real stop codon UAA in yeasts. Comparative analysis indicates that stop codons at this location are considerably more conserved than sense codons, suggesting that these tandem stop codons are maintained by selection. We evaluated the influence of expression levels of genes and other biological factors on the distribution of tandem stop codons. Our results suggest that expression level is an important factor influencing the presence of tandem stop codons.

**Conclusions:**

Our study demonstrates the existence of tandem stop codons, which represent one of many meaningful genomic features that are driven by relatively weak selective forces.

## Abstract 2

This study shows that a statistical excess of stop codons has evolved at the third codon downstream of the real stop codon UAA in yeasts. Comparative analysis indicates that stop codons at this location are considerably more conserved than sense codons, suggesting that these tandem stop codons are maintained by selection.

## Background

In most organisms, one of three stop codons (UAA, UAG and UGA) signals the termination of protein translation. Occasionally a near-cognate tRNA misreads a stop codon and the ribosome reads through the termination signal. Nichols [1] proposed that a second stop codon following the real termination codon could act as a backup. Since this codon follows the real stop codon, it is called a 'tandem stop codon'. By giving the translation machinery a second chance to terminate protein translation [2], tandem stop codons provide a 'fail safe mechanism'.

When read-through occurs, extra amino acids are added to the end of the peptide chain. The presence of tandem stop codons downstream of genes reduces the number of extra amino acids, which would influence protein folding; the addition of fewer extra amino acids increases the likelihood that the protein will preserve its three-dimensional structure. In addition, minimizing the number of amino acids added to the polypeptide chain is also beneficial from a purely energetic point of view. These factors suggest that the existence of tandem stop codons would confer a selective advantage, and imply that such codons do not need to follow the real termination signal immediately to be beneficial.

So far, the existence of tandem stop codons has remained elusive. In *Escherichia coli*, for example, a recent study argued that the slightly over-represented tandem stop codons occur only as a consequence of the strong preference for U at the first nucleotide position following the stop codon as part of an efficient stop signal [3]. Tandem stop codons in eukaryotes have not been rigorously examined. Therefore we decided to analyze tandem stop codons in yeasts. We chose yeast as a model system for two main reasons: first, *Saccharomyces cerevisiae *is the best-studied eukaryotic genome and the quality of its annotation has been greatly improved by recent comparative analyses [4-6], making it possible to detect signals of weak selection forces at the genomic level; second, rich experimental data on translation termination efficiency are available to further characterize the nature of these forces.

In this study we carried out a computational analysis to address the question of whether tandem stop codons occur at any location downstream of yeast genes with unusually high frequency; whether tandem stop codons are under selection; and what is the primary factor influencing tandem stop codons.

## Results

### Are tandem stop codons over-represented in the downstream sequences of yeast genes?

In this study we define tandem stop codons as any stop codon that is downstream from, in the same frame as, and within a relatively short distance from, the real stop codon. We only considered the first nine codon locations downstream of the annotated stop codon, which are all in non-coding regions. The frequency of stop codons in each of these locations following the real stop codon was determined separately for all genes in *S. cerevisiae *that use TAA, TAG, and TGA as stop codons, respectively (throughout this paper T is used instead of U where genomic sequences are considered). The frequency of stop codons at the corresponding locations following each of these three nucleotide triplets (TAA, TAG and TGA) in non-coding regions was also calculated as a control.

**Figure 1:**
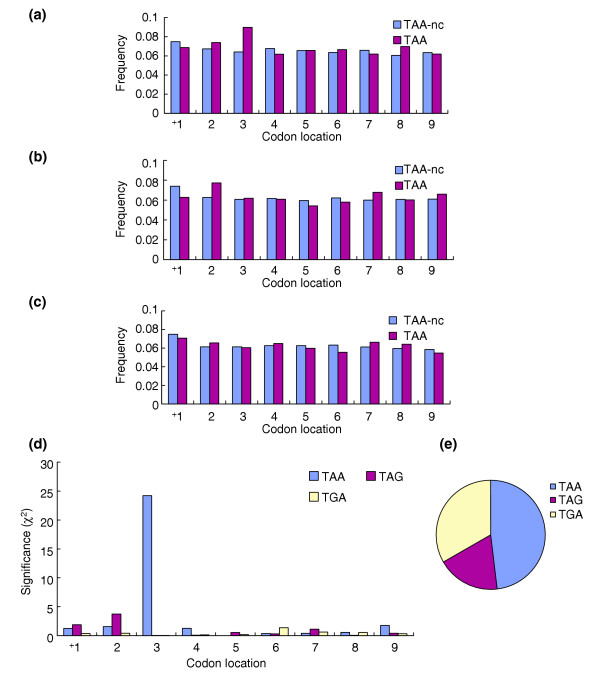
Frequency of stop codons at the downstream codon locations following the real stop codons (which occur at codon location '0') in *S. cerevisiae*. **(a) **TAA; **(b) **TAG; **(c) **TGA. The red bars represent the frequency of stop codons at each codon location and the blue bars represent the controls - the frequency of stop codons at the corresponding locations downstream of stop codons in non-coding regions (nc). **(d) **Statistical significance of stop codon frequency at each location following each stop codon. For each codon location in the analysis, the statistical significance (using χ^2 ^tests) of the difference between the frequencies of tandem stop codons in the yeast genes and in the control sequences is shown. The blue bars represent codon locations following the stop codon TAA; the red bars represent codon locations following the stop codon TAG; the yellow bars represent codon locations following the stop codon TAA. **(e) **The identity distribution of three stop codons at the UAA+3 codon location. Blue represents the percentage of TAA; red represents TAG; yellow, TGA.

These results are shown in Figure 1a-d. Surprisingly, we found that a highly significant excess of stop codons has evolved at the third codon located downstream of the real stop codon TAA, designated here as the UAA+3 codon (we use U instead of T to indicate mRNA) (9.0% versus 6.4%, χ^2 ^= 24.2, *P *< 9 × 10^-7^). No significant excess of stop codons was detected at any other location. The identity of stop codons at the UAA+3 codon location did not matter (Figure 1e); we found no statistical significance of any particular stop codon being either over-represented or under-represented at this site.

**Figure 2:**
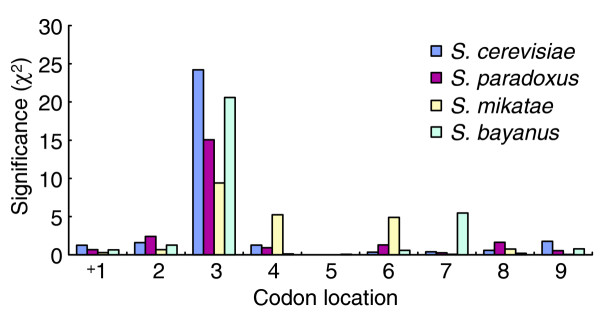
Statistical significance of the frequency of tandem stop codons. Statistical significance of the frequency of tandem stop codons following a TAA stop codon in the first nine codon locations in four *Saccharomyces *species is shown.

We examined this location in three other closely related yeast species, *S. paradoxus*, *S. mikatae *and *S. bayanus*, and in the distantly related yeast *Candida glabrata*, which was recently sequenced [7]. Statistically significant excesses of stop codons were confirmed at this location in all species (*S. paradoxus *χ^2 ^= 15.1, *P *< 1 × 10^-4^,*S. mikatae *χ^2 ^= 9.4, *P *< 2 × 10^-3^; *S. bayanus *χ^2 ^= 20.6, *P *< 6 × 10^-6^; *C. glabrata *χ^2 ^= 9.0, *P *< 3 × 10^-3^) (Figure 2 and Additional data file 1). As another control, we performed the same analysis in the other two frames (frame+2, with codons beginning at the second nucleotide after the stop codon; and frame+3, with codons beginning at the third nucleotide after the stop codon) in *S. cerevisiae *and found several other weakly over-represented locations, such as UAA+1 (frame+2), but none of these trinucleotide locations shows the same tendency in the other species (Additional data files 2 and 3).

In-frame codon UAA+3 is the only location significantly over-represented among all the yeast species, indicating that tandem stop codons at UAA+3 are well conserved in yeasts to a large extent. This conservation in all examined species strongly suggests that the observed excess is biologically meaningful, rather than random noise. Therefore, the following analysis will focus only on tandem stop codons at this location.

### Are tandem stop codons at the UAA+3 codon location under selection?

In order to determine if the excess of tandem stop codons at the UAA+3 codon location are under selection, we examined the third codon following the real stop codon in the 1,029 unambiguous orthologous gene pairs between *S. cerevisiae *and *S. bayanus *(defined in [4]) in which both orthologs use the UAA stop codon. *S. bayanus *was chosen because, among the three *Saccharomyces *species, it is the most distantly related to *S. cerevisiae *[4] and thus the substitutions between these two species provide a better resolution for comparison.

The ancestral states of UAA+3 codons were reconstructed using parsimony. Then the number of conserved stop codons, non-conserved stop codons, conserved sense codons and non-conserved sense codons were calculated and are shown in Table 1. To test whether stop codons at this location are statistically more conserved than sense codons, we used a chi-squared independence test. We found that the conservation of codons at the UAA+3 location strongly depends on whether it is a stop codon (χ^2 ^= 6.1, *P *< 0.01) (Table 1). Furthermore, we performed the same analysis between *S. cerevisiae *and each of the other two remaining *Saccharomyces *species. The tendency of stop codons to be more conserved than sense codons at UAA+3 was the same in all comparisons (between *S. cerevisiae *and *S. paradoxus*, 1,509 orthologous gene pairs, χ^2 ^= 2.8, *P *< 0.1; between *S. cerevisiae *and *S. mikatae*, 1,075 orthologous gene pairs, χ^2 ^= 4.6, *P *< 0.03). With the increase in evolutionary divergence in the comparative analysis, the statistical significance becomes more striking.

In addition, among 504 gene groups in which all the orthologous genes in the four *Saccharomyces *species use UAA as stop codons, 10.5% of them have tandem stop codons at UAA+3, which is much higher than random expectation (χ^2 ^= 18, *P *< 2 × 10^-5^). Therefore, tandem stop codons at the UAA+3 codon location appear to be maintained by selection.

### Does codon bias influence the distribution of tandem stop codons?

We calculated the codon bias (effective number of codons, ENC) [8] of all the genes with UAA stop codons in *S. cerevisiae*. The number of genes with a tandem stop codon located at the UAA+3 location in the high codon bias quartile (25% of genes with the lowest ENC values) and the low codon bias quartile (25% of genes with the highest ENC values) were determined and then compared. To test whether tandem stop codons tend to follow genes with high codon bias, we used a chi-squared independence test. We found that the proportion of genes with a tandem stop codon in the high codon bias quartile is significantly higher than that in the low codon bias quartile (15% versus 6%; χ^2 ^= 27.2, *P *< 2 × 10^-7^, Table 2).

**Figure 3:**
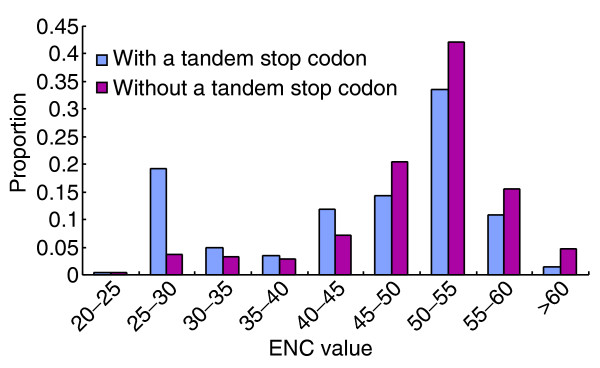
Distribution of tandem stop codons in different codon bias groups in *S. cerevisiae*. The blue bars represent the proportion of genes with a tandem stop codon; the red bars represent the proportion of genes without a tandem stop codon.

A Kolmogrov-Smirnov test also indicated that the codon bias distributions were significantly different (*P *< 2.7 × 10^-9^, Figure 3) between the genes with and without a tandem stop codon.

### Do tandem stop codons tend to occur in essential genes or genes with shared features?

First, we classified the genes with UAA stop codons into four groups (essential genes, strong fitness effect, moderate fitness effect and weak fitness effect) on the basis of the minimum fitness value across five different growth conditions [9]. The fitness values for each media condition were calculated as the extent of survival and reproduction of the deletion strain relative to the pool of all strains grown and measured collectively [10]. The number of genes with tandem stop codons in the essential gene group and weak effect group were determined and then compared. We tested whether tandem stop codons tend to follow biologically important genes using a chi-squared independence test. We found that the proportion of genes with a tandem stop codon in the essential gene group is not significantly different from that in the weak fitness effect group (9.2% versus 8.0%, χ^2 ^= 0.621, *P *= 0.43, Table 3).

Second, we studied the distribution of the genes with tandem stop codons in different biological processes, biological function categories and biological components, respectively (10 Gene Ontology (GO) biological processes, seven GO functional categories and seven GO biological components; see details in Additional data files 4, 5, 6) [11]. In terms of biological processes, genes with tandem stop codons are over-represented in Metabolism (*P *< 0.006) and under-represented in Cell Cycle (*P *< 0.01). In terms of biological functions, genes with tandem stop codons are over-represented in Structural Molecular Activity (*P *< 0.008). In terms of biological components, genes with tandem stop codons are over-represented in the Cytosol (*P *= 0) and Cytoplasm (*P *< 0.01). These observed biases might intrinsically be explained by the difference in expression levels of these specific groups of genes. Third, we studied the distribution of genes with tandem stop codons among different chromosomes. No distribution bias was detected, indicating that the existence of tandem stop codons extends to the whole genome. Fourth, we examined the distribution of genes with tandem stop codons among transcripts with different lengths. No correlation between tandem stop codon frequency and the length of transcripts could be detected, indicating that the length of a transcript has no influence on the presence of a tandem stop codon.

## Discussion

Our results show that a statistically significant excess of stop codons has evolved at the third codon location downstream of UAA stop codons, designated UAA+3, and that this feature is conserved across five distinct yeast species for which data are available. Comparative analysis between closely related species has demonstrated that these stop codons are more conserved than sense codons at the same location, indicating that the tandem stop codons are maintained by selection. While our results support the long-standing hypothesis that tandem stop codons exist, it raises two crucial questions: (i) why is an excess of stop codons observed only in genes using UAA as stop, and not in genes using UAG and UGA? (ii) why do tandem stop codons evolve mainly at the third codon location after UAA, and not the first or second codon locations?

There are several possible answers to the first question. One straightforward explanation is that UAA may be a weak stop codon compared to UAG and UGA, thus requiring a backup stop codon more often. This is not the case: experiments have indicated that UAA is the most efficient termination codon in yeast [12]. Since UAA is the most frequently used stop codon in highly expressed yeast genes [13], another explanation is that tandem stop codons may tend to occur in genes whose products are in high abundance.

To test this hypothesis, we analyzed the correlation between tandem stop codon distribution and codon bias. Codon bias reflects the propensity of an organism to utilize selectively certain codons. Several studies have shown that codon bias is a good indicator of protein abundance, because highly expressed proteins generally have high codon bias [14,15]. Therefore, codon bias can be used as an approximation for protein expression level, although the protein abundance of a gene cannot be predicted specifically based on its codon bias alone [16]. We found that the distribution of tandem stop codons strongly correlates with codon bias, indicating that protein expression level is an important factor influencing tandem stop codons. We further considered the relationship between tandem stop codon distribution and fitness effects of genes, based on the comparison of tandem stop codon frequency between the essential gene group and weak-effect gene group, and found no correlation.

Selection on tandem stop codons is non-negligible in this study only in the highly expressed genes where translation termination occurs many times during the life cycle of yeast. Thus, regardless of the fitness effects of the gene, it seems that tandem stop codons tend to follow frequently used stop codons in the third downstream codon. As a result, it is not surprising that tandem stop codons follow yeast genes that use UAA as a stop and not those that use UAG or UGA, because only a small number of the highly expressed yeast genes use the latter two codons for termination.

Regarding the location of tandem stop codons, it is surprising that tandem stop codons mainly evolve at the third codon location after the real stop codons. The biological reason underlying this observation remains unclear. One possible explanation is that the location of tandem stop codons may be related to the termination context of real stops. Numerous results have indicated that the efficiency of translation termination is influenced by the local context surrounding stop codons [12,17]. Recent studies established that the six nucleotides after the stop codon (corresponding to codon locations +1 and +2 in this study) are key determinants of read-through frequency in yeast, and that these nucleotides can influence read-through efficiency more than 10-fold [18,19]. Thus there may be strong evolutionary constraint on the first two downstream codons to maintain an efficient context for the real stop codon, the primary site for signaling termination. A tandem stop codon may not be favorable at either of these locations, as it may not work well as part of the termination signal. For example, at the first nucleotide following the real UAA stop codon, the most common and the most efficient base for termination is G rather than U [12,17], which precludes this nucleotide from being the first position in a stop codon. This may also explain the under-representation of tandem stop codons at the first codon immediately following the actual stop codon in yeasts (Figure 1). The same bias was also observed in other eukaryotes (see details in additional data files 7 and 8).

As a backup, a tandem stop codon can reduce the negative effects of translation termination errors and would therefore provide a selective advantage. However, this selection should be very weak since translation termination in general is very efficient, and read-through is very rare, with error levels estimated at 0.3% in yeast [18]. Therefore, if the dominant selection on the first two codon locations (the next six nucleotides) following the real stop codon is to maintain a favorable context for efficient translation termination, rather than to accumulate tandem stop codons as backup, the third codon location may become the main site for tandem stop codons.

Tandem stop codons are a relatively subtle regulatory mechanism. While the fitness contribution of a single tandem stop codon may be negligible, the whole effect of over-represented tandem stop codons at the genomic level is probably not. It would be desirable to know whether this (or a similar) mechanism operates in other species. However, the answer is not easy to address at this moment. For most bacteria and archaea, the total number of genes is generally very small. Therefore, it is impossible to perform a similar statistical analysis in these genomes, as the absolute percentage of genes with tandem stop codons is low. Regarding other eukaryotes, we performed the same analysis on *Drosophila melanogaster *and *Caenorhabditis elegans *and found one or two over-represented downstream codons (see Additional data files 7 and 8), but the statistical significance is very weak. Here it should be emphasized that our success in identifying tandem stop codons in yeasts lies on three necessary factors. First and foremost, recent comparative analysis has greatly improved the quality of *Saccharomyces *species genome annotation, leading to removal of about 500 spurious genes and modification of about 10% of the annotated boundaries of coding regions [4]. This allowed us to observe a strong signal over background. Second, the availability of several closely related yeast genomes permits identification of conserved signals and further filters out background noise. Third, the influence of local contexts on termination and read-through efficiency in yeasts has been the subject of intensive experimental study, which is not true for most eukaryotes. Together, these factors limit the ability to expand this study at the present time.

## Conclusion

Our study demonstrates for the first time the existence of tandem stop codons at the genomic level - a long-standing and intriguing hypothesis. Our results indicate that protein expression level is an important biological factor influencing the presence of tandem stop codons. We hope that our study of yeasts will provide a model for future examinations of other groups of species.

## Materials and methods

Gene sequences of four yeast species (*S. cerevisiae*, *S. paradoxus*, *S. mikatae *and *S. bayanus*) were downloaded from [20]. Spurious genes and genes with ambiguous boundaries were excluded from our analysis. Sequences of *C. glabrata *were downloaded from GenBank (NC_005967-005968 and NC_006026-006036).

We used PERL scripts to calculate the frequency of stop codons at the nine codon locations downstream of annotated stop codons. As a control, we used non-coding regions of the same genome: first we looked for occurrence of the trinucleotides TAA, TAG and TGA; then we calculated the frequency of occurrence of these trinucleotides in the same frame at the next nine codons. Statistical significance of the difference between the observed tandem stop codon frequency and the corresponding frequency in the control was determined by chi-squared independence tests. Because we used non-coding DNA sequences of the same genome as a control, factors like single and dinucleotide composition are automatically included in the analysis and do not need to be explicitly considered. Similar analyses were also performed on the other two reading frames for comparison. Because here we studied many codon locations simultaneously and used a less restrictive *P*-value cutoff (0.05), it is very important to examine the signals in all yeast species. We interpret only the codon locations significantly over-represented in all species as biologically meaningful.

Information about one-to-one orthologous gene pairs between *S. cerevisiae *and *S. bayanus *was extracted from [4]. The orthologous gene pairs using UAA as stop codon were used in the conservation analysis. We first reconstructed the ancestral states of UAA+3 codons using a simple parsimony rule. If two orthologous genes share one identical codon at the UAA+3 location, the codon is assumed to be the ancestral state of the UAA+3 codon. If two orthologous genes have different codons at the UAA+3 codon location, each codon is assumed to be the ancestral state with 0.5 probability. Then we compared the inferred ancestral UAA+3 codon with the UAA+3 codon in each of the orthologous genes and decided whether it is conserved in evolution. The number of conserved stop codons, non-conserved stop codons, conserved sense codons and non-conserved sense codons were calculated, respectively. Statistical significance of the conservation difference between stop codons and sense codons was tested by a chi-squared independence test. Here we used a very strict definition of conserved stop codon, requiring it to be identical between the inferred ancestor and its descendant (modern species). Even with this strict criterion, the *P*-value is significant. If we relax the definition to allow any stop codon, the result would be even more significant. The same analysis was carried out between *S. cerevisiae *and *S. paradoxus/S. mikatae*, respectively.

Codon bias (effective number of codons, ENC) of all the genes using UAA as stop codon in *S. cerevisiae *was calculated using the CODONW program [21]. Statistical significance of the proportion of genes with and without tandem stop codons in the genes with the highest 25% ENC values versus the lowest 25% was tested by a chi-squared independence test. The difference in codon bias distribution between these two gene groups (with/without a tandem stop codon) was determined by the Kolmogrov-Smirnov test using MATLAB (version 6.5).

Fitness measurements were obtained from a high-throughput study [9] that measured the growth of each strain of a nearly complete collection of yeast single-gene-deletion mutants under five growth conditions. We calculated the fitness values for growth in each medium condition and then classified all genes using UAA as the stop codon into four groups based on these fitness values (*f*). The calculation of the fitness value and gene classification are the same as in [10]. Statistical significance of the difference in tandem stop codon frequency between the weak-effect gene group (*f *> 0.95 for all five media conditions) and the essential gene groups (if the deletion is lethal) was determined by a chi-squared independence test.

The study of the distribution of genes with tandem stop codons in different biological processes, biological function categories, and biological components was performed by GO Term Mapper at the *Saccharomyces *Genome Database [22]. The set of all genes with UAA stop codons was used as a control to determine whether the set of genes containing tandem stop codons is statistically over-represented or under-represented in a specific gene category.

## Additional data files

The following additional data are available with the online version of this paper. Additional data file 1 gives the frequency of stop codons at each codon location following the real stop codon in other yeast species. Additional data file 2 gives the results for stop codons at each codon location following the real stop codons in all three reading frames in *S. cerevisiae*. Additional data file 3 gives the results of over-represented codon locations in other yeast species. Additional data file 4 gives the distribution of genes with a tandem stop codon in different biological processes in *S. cerevisiae*. Additional data file 5 gives the distribution of genes with a tandem stop codon in different biological functional categories in *S. cerevisiae*. Additional data file 6 gives the distribution of genes with a tandem stop codon in different biological components in *S. cerevisiae*. Additional data file 7 gives frequency of stop codons at each codon location following the real stop codons in *Drosophila melanogaster*. Additional data file 8 gives frequency of stop codons at each codon location following the real stop codons in *Caenorhabditis elegans*. Additional data file 9 lists the genes with a tandem stop codon in different yeast species.

## Supplementary Material

Additional File 1 Table 1, Frequency of stop codons at each codon location following the real stop codons in *S. bayanus*. Table 2, Frequency of stop codons at each codon location following the real stop codons in *S. paradoxus*. Table 3, Frequency of stop codons at each codon location following the real stop codons in *S. mikatae*. Table 4, Frequency of stop codons at each codon location following the real stop codons in *C. glabrata*. Click here for file

Additional File 2 In-frame UAA+3 is the only codon location significantly over-represented in all the yeast species. Click here for file

Additional File 3 Because here we studied many codon locations simultaneously and used a less restrictive *P*-value cutoff, it is very important to further examine the biological signals in other yeast species. Therefore, over-represented locations were examined. Only in frame codon location **UAA+3 **showed the same tendency in all other species. 1. Significant *P*-values are shown in bold (95% confidence level; p < 0.05). 2. Under-represented positions are shown as "n.a.". Click here for file

Additional File 4 The blue bars represent the percentages of genes with a tandem stop codon in each biological process and the red bars represent the controls - the percentages of genes with a TAA stop codon in the corresponding groups. Click here for file

Additional File 5 The red bars represent the percentages of genes with a tandem stop codon in each biological functional category and the red bars represent the controls - the percentages of genes with a TAA stop codon in the corresponding groups. Click here for file

Additional File 6 The blue bars represent the percentages of genes with a tandem stop codon in each biological component and the red bars represent the controls - the percentages of genes with a TAA stop codon in the corresponding groups. Click here for file

Additional File 7 The red bars represent the frequency of stop codons at each codon location and the blue bars represent the controls - the frequency of stop codons at the corresponding locations downstream of stop codons in non-coding regions. Click here for file

Additional File 8 The red bars represent the frequency of stop codons at each codon location and the blue bars represent the controls - the frequency of stop codons at the corresponding locations downstream of stop codons in non-coding regions. Click here for file

Additional File 9 1. List of genes with a tandem stop codon in *S. cerevisiae *2. List of genes with a tandem stop codon in *S. bayanus *3. List of genes with a tandem stop codon in *S. mikatae *4. List of genes with a tandem stop codon in *S. paradoxus *5. List of genes with a tandem stop codon in *C. glabrata* Click here for file

## Figures and Tables

**Table 1:** Frequency of different codon groups at the UAA+3 location in the conservation analysis

	Stop codon	Sense codon	Total
Conserved	138	1,089	1,227
Non-conserved	66	765	831
Total	204	1,854	2,058

χ^2 ^= 6.1; *P *< 0.01.

**Table 2:** The influence of codon bias on the presence of tandem stop codons

	Highest codon bias quartile	Lowest codon bias quartile	Total
With a tandem stop	87	33	120
Without a tandem stop	480	534	1,014
Total	567	567	1,134

χ^2 ^= 27.2; *P *< 2 × 10^-7^.

**Table 3:** The influence of fitness effect of the genes on the presence of tandem stop codons

	Essential genes	Weak-effect genes	Total
With the tandem stop	38	97	135
Without the tandem stop	374	1,118	1,492
Total	412	1,215	1,627

χ^2 ^= 0.621; *P *= 0.43.
