# Supplementary material for: Conservation of tandem stop codons in yeasts
Source: Genome Biol. 2005 Mar 15;6(4):R31. doi: 10.1186/gb-2005-6-4-r31 (PMC1088959; doi:10.1186/gb-2005-6-4-r31)
Supplement: Additional File 1 — Table 1, Frequency of stop codons at each codon location following the real stop codons in S. bayanus. Table 2, Frequency of stop codons at each codon location following the real stop codons in S. paradoxus. Table 3, Frequency of stop codons at each codon location following the real stop codons in S. mikatae. Table 4, Frequency of stop codons at each codon location following the real stop codons in C. glabrata. [file gb-2005-6-4-r31-S1.pdf]

Additional Data file 1

Frequency of stop codons at each codon location following the real stop codons in other yeast species.

Table 1 Frequency of stop codons at each codon location following the real stop codons in *S. bayanus.*

Frequency/	TAA		TAG		TGA	
Position	Observed	Control	Observed	Control	Observed	Control
Codon +1	0.0688	0.0737	0.0479	0.0697	0.0781	0.0624
Codon +2	0.0579	0.0644	0.0527	0.0610	0.0606	0.0578
Codon +3	0.0854	0.0599	0.0732	0.0600	0.0535	0.0588
Codon +4	0.0668	0.0649	0.0499	0.0576	0.0511	0.0585
Codon +5	0.0607	0.0621	0.0607	0.0574	0.0536	0.0576
Codon +6	0.0635	0.0592	0.0509	0.0580	0.0552	0.0572
Codon +7	0.0735	0.0603	0.0480	0.0572	0.0472	0.0589
Codon +8	0.0625	0.0600	0.0627	0.0540	0.0553	0.0572
Codon +9	0.0631	0.0582	0.0697	0.0569	0.0513	0.0591

Table 2 Frequency of stop codons at each codon location following the real stop codons in *S. paradoxus.*

Frequency/	TAA		TAG		TGA	
Position	Observed	Control	Observed	Control	Observed	Control
Codon +1	0.0724	0.0772	0.0577	0.0735	0.0821	0.0673
Codon +2	0.0753	0.0667	0.0633	0.0618	0.0575	0.0610
Codon +3	0.0853	0.0643	0.0699	0.0639	0.0618	0.0597
Codon +4	0.0696	0.0644	0.0540	0.0618	0.0626	0.0601
Codon +5	0.0649	0.0649	0.0597	0.0582	0.0655	0.0597
Codon +6	0.0672	0.0613	0.0550	0.0596	0.0619	0.0592
Codon +7	0.0587	0.0614	0.0664	0.0605	0.0619	0.0582
Codon +8	0.0707	0.0638	0.0769	0.0615	0.0626	0.587
Codon +9	0.0645	0.0606	0.0608	0.0601	0.0597	0.0584

Table 3 Frequency of stop codons at each codon location following the real stop codons in *S. mikatae.*

Frequency/ Position	TAA		TAG		TGA	
	Observed	Control	Observed	Control	Observed	Control
Codon +1	0.0798	0.0764	0.0602	0.0795	0.0596	0.0713
Codon +2	0.0651	0.0725	0.0729	0.0643	0.0655	0.0634
Codon +3	0.0832	0.0651	0.0824	0.0662	0.0597	0.0597
Codon +4	0.0544	0.0683	0.0667	0.0632	0.0673	0.0629
Codon +5	0.0669	0.0667	0.0562	0.0627	0.0581	0.0626
Codon +6	0.0777	0.0646	0.0710	0.0653	0.0682	0.0624
Codon +7	0.0630	0.0647	0.0636	0.0622	0.0658	0.0606
Codon +8	0.0693	0.0642	0.0605	0.0615	0.0684	0.0616
Codon +9	0.0647	0.0635	0.0605	0.0619	0.0668	0.0593

Table 4 Frequency of stop codons at each codon location following the real stop codons in *C. glabrata.*

Frequency/ Position	TAA		TAG		TGA	
	Observed	Control	Observed	Control	Observed	Control
Codon +1	0.0720	0.0926	0.0483	0.0594	0.0786	0.0779
Codon +2	0.0771	0.0732	0.0622	0.0688	0.0736	0.0682
Codon +3	0.0916	0.0747	0.0786	0.0731	0.0872	0.0680
Codon +4	0.0822	0.0738	0.0737	0.0711	0.0543	0.0683
Codon +5	0.0783	0.0725	0.0590	0.0697	0.0672	0.0711
Codon +6	0.0790	0.0729	0.0934	0.0753	0.0643	0.0701
Codon +7	0.0853	0.0735	0.0647	0.0658	0.0808	0.0687
Codon +8	0.0814	0.0705	0.0680	0.0684	0.0715	0.0691
Codon +9	0.0735	0.0695	0.0737	0.0719	0.0629	0.0642
