# Supplementary material for: Conservation of tandem stop codons in yeasts
Source: Genome Biol. 2005 Mar 15;6(4):R31. doi: 10.1186/gb-2005-6-4-r31 (PMC1088959; doi:10.1186/gb-2005-6-4-r31)
Supplement: Additional File 2 — In-frame UAA+3 is the only codon location significantly over-represented in all the yeast species. [file gb-2005-6-4-r31-S2.pdf]

Additional Data file 2

Frequency of tandem stop codons in all three reading frames following the real stop codons in *S. cerevisiae.* In-frame UAA+3 is the only codon location significantly over-represented in all the yeast species.

Frequency of tandem stop codons in all three reading frames following the real stop codon UAA.

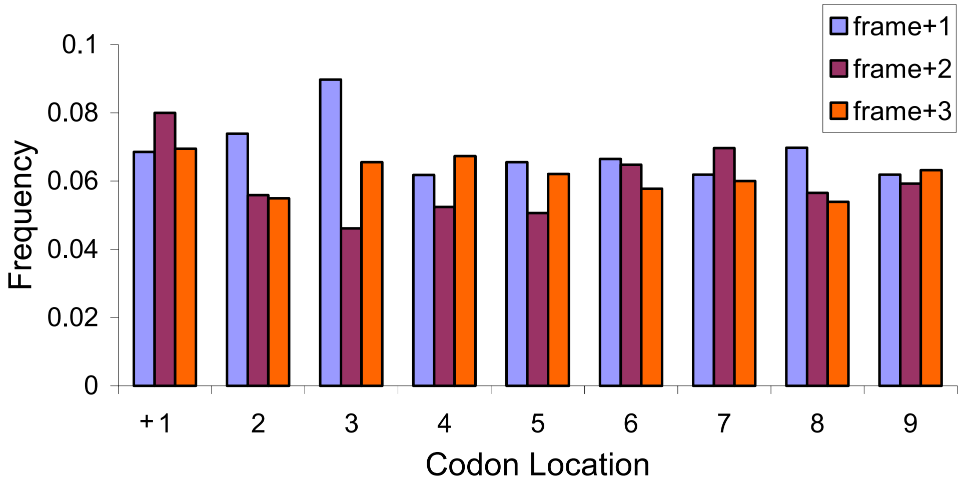

Frequency of tandem stop codons in all three reading frames following the real stop codon UGA.

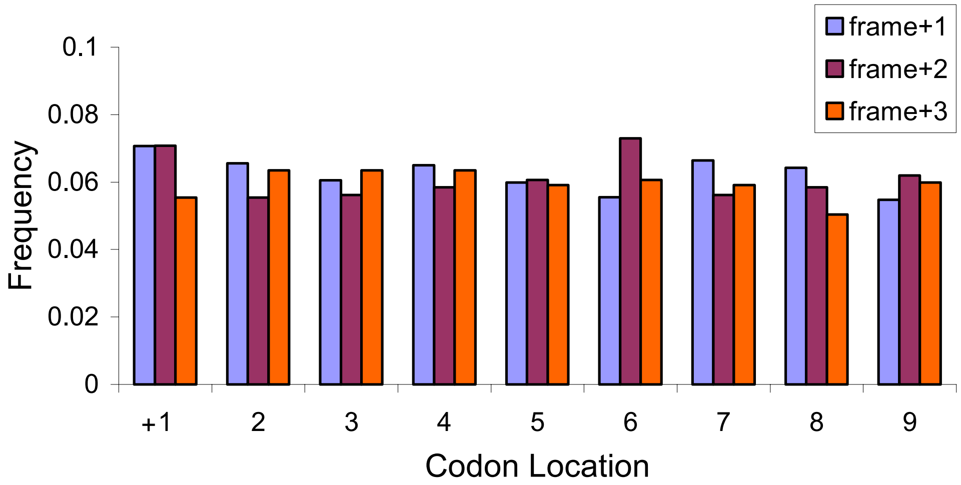

Frequency of tandem stop codons in all three reading frames following the real stop codon UAG.

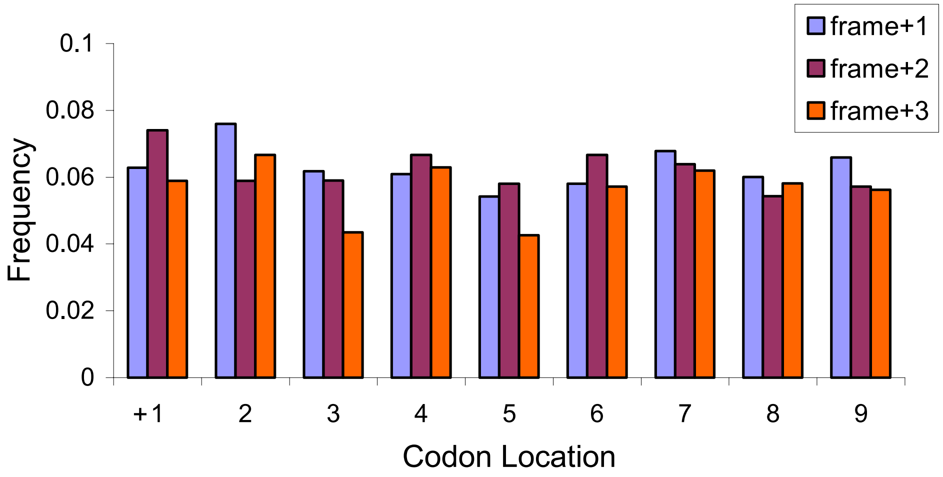

Statistical significance of frequency of tandem stop codons in all three reading frames following the real stop codons in *S. cerevisiae.*
1. Significant P-values are shown in bold (95% confidence level; $p < 0.05$)
2. Under-represented positions are shown as "n.a.".

S. cerevisiae	UAA	UAG	UGA
	P-value	P-value	P-value
Frame+1			
Position1	n.a.	n.a.	n.a.
Position2	0.2	0.053	0.5
Position3	**9E-07**	0.9	n.a.
Position4	n.a.	n.a.	0.7
Position5	1	n.a.	n.a.
Position6	0.6	n.a.	n.a.
Position7	n.a.	0.3	0.4
Position8	n.a.	n.a.	0.5
Position9	0.07	0.5	n.a.
Frame+2			
Position1	**0.005**	0.2	**0.02**
Position2	n.a.	n.a.	n.a.
Position3	n.a.	n.a.	n.a.
Position4	n.a.	0.2	n.a.
Position5	n.a.	n.a.	0.7
Position6	0.8	0.4	0.06
Position7	0.07	0.6	n.a.
Position8	n.a.	n.a.	n.a.
Position9	n.a.	n.a.	0.9
Frame+3			
Position1	0.2	n.a.	n.a.
Position2	n.a.	0.6	n.a.
Position3	0.8	n.a.	0.8
Position4	0.5	0.6	n.a.
Position5	0.9	n.a.	n.a.
Position6	n.a.	n.a.	0.6
Position7	n.a.	0.7	n.a.
Position8	n.a.	n.a.	n.a.
Position9	0.5	n.a.	n.a.
