# Supplementary material for: Conservation of tandem stop codons in yeasts
Source: Genome Biol. 2005 Mar 15;6(4):R31. doi: 10.1186/gb-2005-6-4-r31 (PMC1088959; doi:10.1186/gb-2005-6-4-r31)
Supplement: Additional File 3 — Because here we studied many codon locations simultaneously and used a less restrictive P-value cutoff, it is very important to further examine the biological signals in other yeast species. Therefore, over-represented locations were examined. Only in frame codon location UAA+3 showed the same tendency in all other species. 1. Significant P-values are shown in bold (95% confidence level; p < 0.05). 2. Under-represented positions are shown as "n.a.". [file gb-2005-6-4-r31-S3.pdf]

Additional Data file 3

Because here we studied many codon locations simultaneously and used a less restrictive P-value cut-off, it is very important to further examine the biological signals in other yeast species. Therefore, over-represented locations were examined. Only in frame codon location **UAA+3** showed the same tendency in all other species.

1. Significant P-values are shown in bold (95% confidence level; $p < 0.05$).
2. Under-represented positions are shown as “n.a.”.

		S.cerevisiae	*S.bayanus*	*S.mikatae*	*S.paradoxus*	*C. glabrata*
		P-value	P-value	P-value	P-value	P-value
**UAA+3**	Frame+1	**9E-07**	**6E-06**	**2E-03**	**1E-04**	**3E-03**
UAG+2	Frame+1	0.053	n.a.	0.3	0.9	0.5
UAA+1	Frame+2	**0.005**	0.5	0.1	**0.013**	0.6
UGA+1	Frame+2	**0.02**	n.a.	0.2	0.3	0.9
