# Supplementary material for: Conservation of tandem stop codons in yeasts
Source: Genome Biol. 2005 Mar 15;6(4):R31. doi: 10.1186/gb-2005-6-4-r31 (PMC1088959; doi:10.1186/gb-2005-6-4-r31)

Additional Data file 6

Distribution of genes with a tandem stop codon in different biological components in *S. cerevisiae*. The blue bars represent the percentages of genes with a tandem stop codon in each biological component and the red bars represent the controls – the percentages of genes with a TAA stop codon in the corresponding groups.

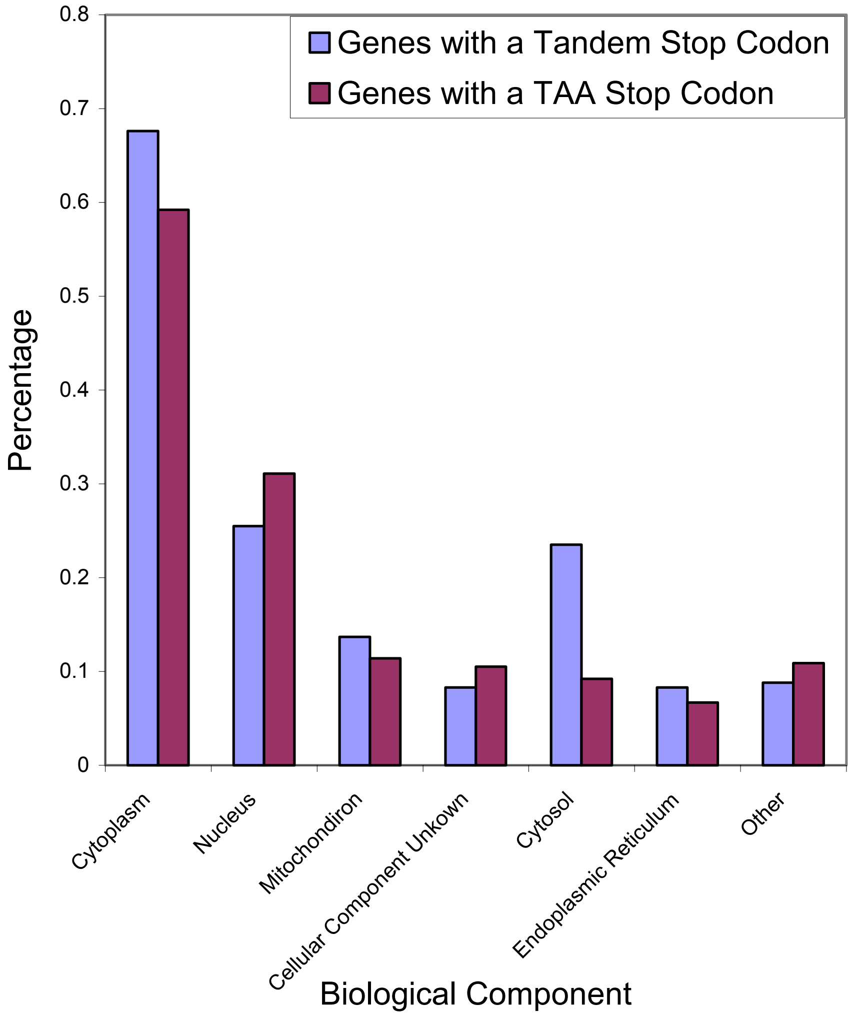

Supplement: Additional File 6 — The blue bars represent the percentages of genes with a tandem stop codon in each biological component and the red bars represent the controls - the percentages of genes with a TAA stop codon in the corresponding groups. [file gb-2005-6-4-r31-S6.pdf]
