# Supplementary material for: Conservation of tandem stop codons in yeasts
Source: Genome Biol. 2005 Mar 15;6(4):R31. doi: 10.1186/gb-2005-6-4-r31 (PMC1088959; doi:10.1186/gb-2005-6-4-r31)

Additional Data file 8

Frequency of stop codons at each codon location following the real stop codons (a) TAA, (b) TAG and (c) TGA in *Caenorhabditis elegans*. The red bars represent the frequency of stop codons at each codon location and the blue bars represent the controls – the frequency of stop codons at the corresponding locations downstream of stop codons in non-coding regions.

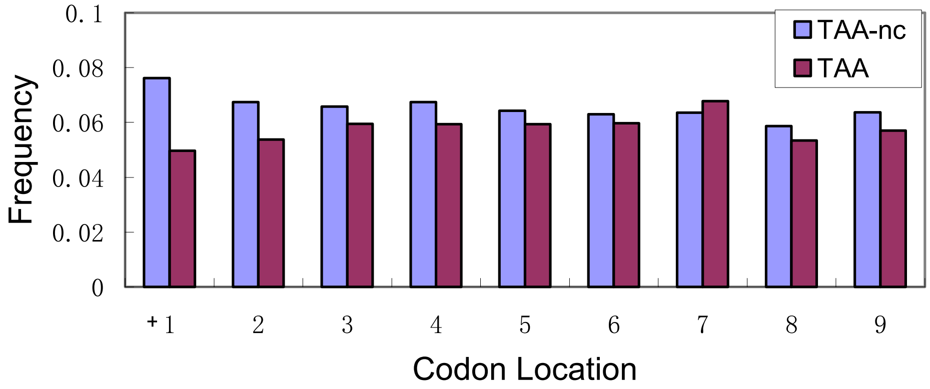

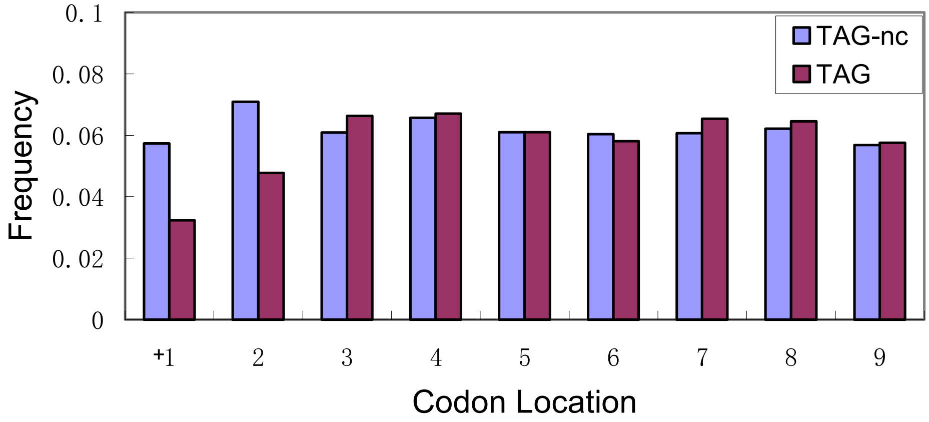

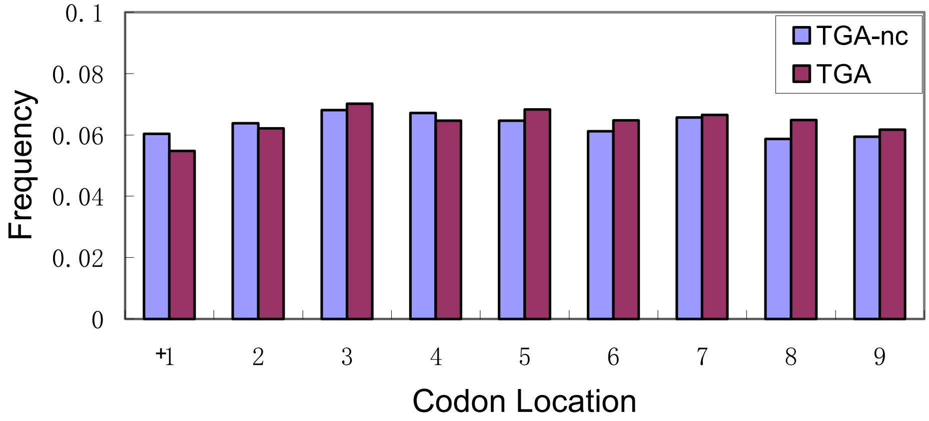

Supplement: Additional File 8 — The red bars represent the frequency of stop codons at each codon location and the blue bars represent the controls - the frequency of stop codons at the corresponding locations downstream of stop codons in non-coding regions. [file gb-2005-6-4-r31-S8.pdf]
