# Supplementary material for: Conservation of tandem stop codons in yeasts
Source: Genome Biol. 2005 Mar 15;6(4):R31. doi: 10.1186/gb-2005-6-4-r31 (PMC1088959; doi:10.1186/gb-2005-6-4-r31)
Supplement: Additional File 9 — 1. List of genes with a tandem stop codon in S. cerevisiae 2. List of genes with a tandem stop codon in S. bayanus 3. List of genes with a tandem stop codon in S. mikatae 4. List of genes with a tandem stop codon in S. paradoxus 5. List of genes with a tandem stop codon in C. glabrata [file gb-2005-6-4-r31-S9.pdf]

Additional Data file 9

Lists of genes with a tandem stop codon in different yeast species.

1. List of genes with a tandem stop codon in *S. cerevisiae*
2. List of genes with a tandem stop codon in *S. bayanus*
3. List of genes with a tandem stop codon in *S. mikatae*
4. List of genes with a tandem stop codon in *S. paradoxus*
5. List of genes with a tandem stop codon in *C. glabrata*

List of genes with a tandem stop codon in *S. cerevisiae*

YAL015C YBL092W YBL045C YBR009C YBR022W YBR026C YBR031W
YBR033W YBR054W YBR118W YBR153W YBR160W YBR166C YBR176W
YBR183W YBR242W YBR259W YBR285W YCL048W YCR014C YCR044C
YCR059C YDL237W YDL233W YDL218W YDL194W YDL192W YDL171C
YDL136W YDL133C-A YDL105W YDL075W YDL021W YDL014W YDR086C
YDR090C YDR099W YDR105C YDR120C YDR229W YDR234W YDR292C
YDR298C YDR302W YDR382W YDR384C YDR410C YDR447C YDR524C
YEL050C YEL034W YER013W YER019C-A YER061C YER074W YER131W
YER132C YER177W YFR024C-A YFR029W YFR032C-A YGL190C YGL189C
YGL186C YGL076C YGL060W YGL030W YGL028C YGL021W YGL008C
YGR086C YGR126W YGR130C YGR148C YGR162W YGR214W YGR217W
YGR254W YHR007C YHR017W YHR027C YHR078W YHR089C YHR124W
YHR179W YHR203C YHR208W YIL142W YIL127C YIL123W YIL116W
YIL042C YIL018W YIR023W YJL203W YJL201W YJL180C YJR010W
YJR011C YJR086W YJR104C YJR118C YJR132W YJR145C YKL188C
YKL129C YKL120W YKL081W YKR055W YKR081C YKR082W YKR095W
YLL050C YLL036C YLL021W YLR022C YLR029C YLR038C
YLR044C YLR061W YLR081W YLR134W YLR209C YLR249W YLR258W
YLR272C YLR286C YLR287C-A YLR292C YLR319C YLR325C YLR344W
YLR347C YLR385C YLR452C YML115C YML110C YML083C YML063W
YML026C YML008C YMR029C YMR054W YMR055C YMR060C YMR078C
YMR099C YMR105C YMR113W YMR131C YMR142C YMR162C YMR258C
YNL320W YNL308C YNL272C YNL253W YNL213C YNL192W YNL188W
YNL182C YNL175C YNL131W YNL069C YNL016W YNR016C YNR064C
YOL148C YOL120C YOL077C YOL058W YOL040C YOL012C YOR064C
YOR117W YOR130C YOR133W YOR137C YOR232W YOR234C YOR244W
YOR298W YOR299W YOR369C YOR375C YPL262W YPL249C-A YPL215W
YPL203W YPL188W YPL143W YPL085W YPL081W YPL075W YPL037C
YPR020W YPR026W YPR033C YPR057W YPR080W YPR102C YPR183W
YPR185W YPR193C

List of genes with a tandem stop codon in *S. bayanus*

YAL001C YBL092W YBL039C YBL011W YBR048W YBR061C YBR118W YBR211C YBR291C YCR016W YCR044C YCR086W YDL230W YDL227C YDL175C YDL149W YDL130W YDL101C YDL090C YDL059C YDL014W YDR006C YDR032C YDR050C YDR052C YDR086C YDR097C YDR127W YDR147W YDR171W YDR179C YDR221W YDR234W YDR258C YDR301W YDR302W YDR356W YDR385W YDR386W YDR447C YDR449C YDR473C YDR519W YEL050C YEL026W YER044C YER047C YER049W YER091C YER136W YFR031C YGL189C YGL160W YGL135W YGL114W YGL103W YGR110W YGR148C YGR162W YGR214W YGR235C YGR264C YGR277C YHR007C YHR094C YHR124W YHR134W YHR143W-A YHR156C YHR170W YHR203C YIL155C YIL142W YIL111W YIL018W YIR003W YIR026C YJL209W YJL201W YJL111W YJL097W YJL051W YJL024C YJL010C YJR003C YJR010W YJR069C YJR145C YJR151C YKL172W YKL170W YKL151C YKL129C YKL082C YKL001C YKR001C YKR010C YKR082W YLL004W YLR029C YLR044C YLR048W YLR074C YLR134W YLR166C YLR167W YLR249W YLR275W YLR298C YLR324W YLR335W YLR340W YLR347C YLR392C YLR409C YLR433C YLR438W YLR452C YML104C YML093W YML063W YML026C YMR033W YMR099C YMR131C YMR183C YMR307W YNL211C YNL099C YNL069C YNR019W YNR035C YOL102C YOL040C YOL019W YOL014W YOL003C YOR093C YOR096W YOR113W YOR117W YOR234C YOR281C YOR287C YOR338W YOR369C YOR375C YOR377W YPL145C YPL120W YPL108W YPL096W YPL070W YPL037C YPR080W

List of genes with a tandem stop codon in *S. mikatae*

YAL001C YAR002C-A YBL045C YBR031W YBR056W YBR061C YBR118W YBR143C YBR160W YBR161W YBR165W YBR215W YBR273C YCR020W-B YCR059C YCR073W-A YDL198C YDL104C YDL102W YDL075W YDL064W YDL001W YDR120C YDR173C YDR179C YDR229W YDR234W YDR298C YDR302W YDR382W YDR384C YDR422C YDR485C YDR486C YER004W YER019C-A YER044C YER091C YER105C YER150W YFR001W YFR004W YFR032C-A YFR046C YGL242C YGL198W YGL190C YGL189C YGL176C YGL166W YGL125W YGL086W YGL080W YGL076C YGL056C YGL008C YGL001C YGR078C YGR156W YGR210C YGR214W YGR221C YGR244C YGR250C YHL011C YHR007C YHR017W YHR027C YHR051W YHR078W YHR089C YHR094C YHR170W YHR176W YIL158W YIL142W YIL135C YIL133C YIL123W YIL076W YIL067C YIL018W YIR022W YJL201W YJL193W YJL179W YJL159W YJR070C YJR086W YJR145C YKL120W YKL088W YKL082C YKL052C YKR008W YKR081C YKR082W YKR100C YLL050C YLL049W YLL048C YLR022C YLR204W YLR249W YLR325C YLR401C YLR447C YLR449W YML075C YML063W YML049C YML031W YMR091C YMR135C YMR225C YMR267W YMR301C YNL329C YNL326C YNL272C YNL242W YNL208W YNL204C YNL147W YNL130C YNL069C YNR016C YNR024W YNR057C YOL148C YOL102C YOL040C YOR117W YOR133W YOR145C YOR232W YOR234C YOR322C YOR348C YOR375C YPL249C-A YPL203W YPL188W YPL145C YPR066W YPR080W YPR183W

List of genes with a tandem stop codon in *S. paradoxus*

YBL092W YBL086C YBL071W-A YBL066C YBL064C YBL006C YBR009C YBR010W YBR023C YBR026C YBR031W YBR054W YBR084W YBR108W YBR118W YBR137W YBR143C YBR148W YBR165W YBR176W YBR183W YBR259W YCL048W YCL043C YCR017C YCR059C YDL237W YDL233W YDL218W YDL204W YDL194W YDL182W YDL171C YDL132W YDL105W YDL058W YDL056W YDL021W YDL014W YDR099W YDR105C YDR120C YDR128W YDR131C YDR232W YDR298C YDR302W YDR311W YDR365C YDR384C YDR447C YDR485C YEL034W YER019C-A YER130C YER132C YFR001W YFR032C-A YGL190C YGL189C YGL103W YGL062W YGL060W YGL040C YGL008C YGL001C YGR007W YGR015C YGR082W YGR090W YGR102C YGR131W YGR148C YGR162W YGR208W YGR214W YGR236C YGR254W YHL032C YHR007C YHR017W YHR041C YHR051W YHR065C YHR078W YHR091C YHR094C YHR101C YHR170W YHR203C YIL145C YIL142W YIL133C YIL123W YIL094C YIL018W YIR023W YJL201W YJL144W YJL105W YJL010C YJR010W YJR086W YJR104C YJR145C YKL188C YKL120W YKL081W YKR021W YKR081C YKR082W YLL009C YLR022C YLR029C YLR044C YLR134W YLR196W YLR249W YLR272C YLR275W YLR287C-A YLR325C YLR347C YLR385C YML063W YML004C YMR055C YMR099C YMR100W YMR111C YMR121C YMR131C YMR197C YMR224C YMR279C YNL272C YNL264C YNL254C YNL225C YNL216W YNL213C YNL202W YNL192W YNL188W YNL131W YNL130C YNL083W YNR008W YNR016C YOL148C YOL127W YOL058W YOL040C YOR093C YOR107W YOR117W YOR133W YOR137C YOR234C YOR244W YOR274W YOR287C YOR298W YOR361C YOR369C YOR375C YPL249C-A YPL203W YPL173W YPL163C YPL145C YPL085W YPL037C YPL019C YPL001W YPR033C YPR066W YPR080W YPR183W

List of genes with a tandem stop codon in *C. glabrata*

GeneID:2886465	GeneID:2886362	GeneID:2886453	GeneID:2886382
GeneID:2886373	GeneID:2886470	GeneID:2886326	GeneID:2886486
GeneID:2886385	GeneID:2886275	GeneID:2886444	GeneID:2886292
GeneID:2886354	GeneID:2886602	GeneID:2886576	GeneID:2886539
GeneID:2886603	GeneID:2886545	GeneID:2886495	GeneID:2886656
GeneID:2886628	GeneID:2886810	GeneID:2886834	GeneID:2886802
GeneID:2886863	GeneID:2886767	GeneID:2886771	GeneID:2886871
GeneID:2886776	GeneID:2886952	GeneID:2886938	GeneID:2887084
GeneID:2887162	GeneID:2887031	GeneID:2887011	GeneID:2887185
GeneID:2887184	GeneID:2887217	GeneID:2887107	GeneID:2887059
GeneID:2887502	GeneID:2887393	GeneID:2887369	GeneID:2887391
GeneID:2887274	GeneID:2887285	GeneID:2887334	GeneID:2887373
GeneID:2887289	GeneID:2887447	GeneID:2887436	GeneID:2887384
GeneID:2887294	GeneID:2887699	GeneID:2887872	GeneID:2887786
GeneID:2887961	GeneID:2887974	GeneID:2887769	GeneID:2887686
GeneID:2887920	GeneID:2887723	GeneID:2887622	GeneID:2887779
GeneID:2887568	GeneID:2887575	GeneID:2888265	GeneID:2888316
GeneID:2888270	GeneID:2888247	GeneID:2887990	GeneID:2888331
GeneID:2888376	GeneID:2888067	GeneID:2888106	GeneID:2888109
GeneID:2888226	GeneID:2888357	GeneID:2888084	GeneID:2888041
GeneID:2888043	GeneID:2888036	GeneID:2888391	GeneID:2888156
GeneID:2888692	GeneID:2888460	GeneID:2888831	GeneID:2888879
GeneID:2888596	GeneID:2888584	GeneID:2888848	GeneID:2888791
GeneID:2888671	GeneID:2888770	GeneID:2888765	GeneID:2888591
GeneID:2888469	GeneID:2888659	GeneID:2888677	GeneID:2888530
GeneID:2889308	GeneID:2889300	GeneID:2889044	GeneID:2889041
GeneID:2888910	GeneID:2889332	GeneID:2888956	GeneID:2889382
GeneID:2889384	GeneID:2889387	GeneID:2889022	GeneID:2889024
GeneID:2889148	GeneID:2889360	GeneID:2889085	GeneID:2889197
GeneID:2889131	GeneID:2889066	GeneID:2889061	GeneID:2889189
GeneID:2889217	GeneID:2888972	GeneID:2888978	GeneID:2889230
GeneID:2889279	GeneID:2889400	GeneID:2889882	GeneID:2889817
GeneID:2889795	GeneID:2889760	GeneID:2889736	GeneID:2889740
GeneID:2889725	GeneID:2889688	GeneID:2889644	GeneID:2889651
GeneID:2889637	GeneID:2889556	GeneID:2889558	GeneID:2889564
GeneID:2889503	GeneID:2889769	GeneID:2889781	GeneID:2889620
GeneID:2889631	GeneID:2889575	GeneID:2889858	GeneID:2889601
GeneID:2889602	GeneID:2889485	GeneID:2889489	GeneID:2889468
GeneID:2890190	GeneID:2890514	GeneID:2890030	GeneID:2890516
GeneID:2890039	GeneID:2889990	GeneID:2890502	GeneID:2890491
GeneID:2890477	GeneID:2890455	GeneID:2890435	GeneID:2890410
GeneID:2890388	GeneID:2890383	GeneID:2890341	GeneID:2890295
GeneID:2890213	GeneID:2890119	GeneID:2890108	GeneID:2890530
GeneID:2889970	GeneID:2889987	GeneID:2890423	GeneID:2891108

GeneID:2890935	GeneID:2890941	GeneID:2890920	GeneID:2890678
GeneID:2890854	GeneID:2890726	GeneID:2890773	GeneID:2890704
GeneID:2890707	GeneID:2890723	GeneID:2890812	GeneID:2890752
GeneID:2890753	GeneID:2890654	GeneID:2890991	GeneID:2890996
GeneID:2890559	GeneID:2891122	GeneID:2891032	GeneID:2890957
GeneID:2890961	GeneID:2890944	GeneID:2890700	GeneID:2890599
GeneID:2890588	GeneID:2891148	GeneID:2891604	GeneID:2891191
GeneID:2891482	GeneID:2891506	GeneID:2891621	GeneID:2891569
GeneID:2891632	GeneID:2891636	GeneID:2891652	GeneID:2891659
GeneID:2891666	GeneID:2891673	GeneID:2891678	GeneID:2891701
GeneID:2891736	GeneID:2891742	GeneID:2891745	GeneID:2891260
GeneID:2891279	GeneID:2891290	GeneID:2891303	GeneID:2891317
GeneID:2891323	GeneID:2891334	GeneID:2891348	GeneID:2891374
GeneID:2891394	GeneID:2891450	GeneID:2891198	GeneID:2891211
GeneID:2891234			
